# A look at risk factors of proteinuria in subjects without impaired renal filtration function in a general population in Owerri, Nigeria

**DOI:** 10.11604/pamj.2016.23.257.8189

**Published:** 2016-04-28

**Authors:** Ernest Ndukaife Anyabolu, Innocent Ijezie Chukwuonye, Arthur Ebelenna Anyabolu, Okezie Enwere

**Affiliations:** 1 Division of Nephrology, Department of Medicine, lmo State University Teaching Hospital, Orlu, Nigeria; 2 Division of Nephrology, Department of Medicine, Federal Medical Centre, Umuahia, Nigeria; 3 Division of Respirology, Department of Medicine, Nnamdi Azikiwe University Teaching Hospital, Nnewi, Nigeria

**Keywords:** Risk factors, proteinuria, urine creatinine, urine osmolality, Owerri, Nigeria

## Abstract

**Introduction:**

Proteinuria is a common marker of kidney damage. This study aimed at determining predictors of proteinuria in subjects without impaired renal filtration function in Owerri, Nigeria.

**Methods:**

This was a cross-sectional study involving 136 subjects, consecutively drawn from Federal Medical Centre (FMC), Owerri, Nigeria. Relevant investigations were performed, including 24-hour urine protein (24HUP). Correlation and multivariate linear regression analysis were used to determine the association and strength of variables to predict proteinuria. Proteinuria was defined as 24HUP ≥0.300g and impaired renal filtration function as creatinine clearance (ClCr) <90mls/min. P<0.05 was taken as statistically significant.

**Results:**

Mean age of subjects was 38.58 ±11.79 years. Female/male ratio was 3:1. High 24-hour urine volume (24HUV) (p<0.001), high spot urine protein/creatinine ratio (SUPCR) (p<0.001), high 24-hour urine protein/creatinine ratio (24HUPCR) (p<0.001), high 24-hour urine protein/osmolality ratio (24HUPOR) (p<0.001), low 24-hour urine creatinine/osmolality ratio (24HUCOR) (p<0.001), and low spot urine protein/osmolality ratio (SUPOR) (p<0.001), predicted proteinuria in this study.

**Conclusion:**

The risk factors of proteinuria in subjects without impaired renal filtration function in Owerri, Nigeria, included 24HUV, SUPCR, 24HUPCR, 24HUPOR, 24HUCOR and SUPOR. Further research should explore the relationship between urine creatinine and urine osmolality, and how this relationship may affect progression of kidney damage, with or without impaired renal filtration function.

## Introduction

The world prevalence of proteinuria in the general population is not known. However, in Australia, a large-scale study showed a proteinuria prevalence of 2.4% in the general population [1]. In USA, prevalence of 1.7% was documented in a study [2]. A prevalence of 4.4% was reported in Japan [3]. Studies from Sub-Saharan Africa showed a similar prevalence [4]. In two studies, Nigeria reported 29.7% and 1.9% [5, 6].

Proteinuria is an established marker of chronic kidney disease. A meta-analysis of studies on chronic kidney disease (CKD) noted that proteinuria was used to determine the presence of kidney damage in only 69% of the studies, while estimated glomerular filtration rate (GFR) was used in the remaining 31% [4]. This has undermined identification and monitoring of patients with CKD who may have chronic kidney damage without impaired GFR.

In the setting of CKD, with or without impaired GFR, proteinuria is a recognized independent risk factor for cardiovascular and renal disease, and a predictor of end-organ damage [7, 8].

The predictors of proteinuria from previous studies included HIV infection, hepatitis C virus infection [9, 10].

There is paucity of studies on the predictors of proteinuria in Nigeria, and none from literature search in the South eastern part of Nigeria. We have therefore, set out to determine the predictors of isolated proteinuria in the general population in Owerri, Nigeria. This will help in identifying potential patients in the general population who may have kidney damage, without impairment of renal filtration function.

## Methods

This was a two-month, cross sectional study conducted in FMC Owerri, in 2011.

One hundred and thirty-six, 18-65 years-old subjects were consecutively recruited from the Medical Out-Patient Department of the hospital.

Approval for this study was obtained from the Research Ethical Committee of FMC.

Informed consent was obtained from all the subjects who took part in this study.

Subjects with kidney disease, diabetes mellitus, hypertension, or any conditions known to be associated with kidney damage and those on nephrotoxic drugs were excluded from the study. Demographic and anthropometric data were collected with use of questionnaire.

Investigations done on each of the subjects were serum creatinine, spot urine protein (SUP), spot urine creatinine (SUCr), spot urine osmolality (SUOsm), 24HUCr, 24-hour urine osmolality (24HUOsm), 24-hour urine protein (24HUP), fasting serum cholesterol, low density lipoprotein cholesterol (LDL), high density lipoprotein cholesterol (HDL), triglyceride.

Creatinine was determined by modified Jeffe's method, protein by photometric method and osmolality by freezing point depression method using Precision System Osmette 5002 osmometer. Creatinine clearance (ClCr), SUPCR, SUPOR, 24HUPCR, 24HUPOR, spot urine creatinine/osmolality ratio (SUCOR), 24HUCOR were determined.

Proteinuria was defined as 24HUP ≥ 0.300g and impaired renal filtration function as ClCr <90mls/min.

Potential risk factors of proteinuria evaluated, here, were: age, serum creatinine, SUP, SUCr, SUOsm, 24HUV, 24HUCr, 24HUOsm, SUPCR, SUPOR, 24HUPCR, 24HUPOR, SUCOR, 24HUCOR, ClCr, body mass index (BMI), waist circumference (WC), cholesterol, LDL, HDL, triglyceride, hemoglobin, systolic blood pressure (SBP), diastolic blood pressure (DBP).

SPSS version 17 was used to analyze the data. The distribution and characterization of variables between subjects with proteinuria and those without proteinuria were determined using cross tabulation. Correlation statistics were used to determine the association of variables with proteinuria, while multivariate linear regression analysis was used to determine the strength of variables to predict proteinuria. P<0.05 was taken as statistically significant.

## Results

The total number of subjects that took part in the study was 136. Females were 98 (72.1%) while males were 38(27.9%). The female/male ratio was 3:1. The mean age of subjects was 38.58±11.79 years. The mean serum creatinine was 0.88±0.19g/dl, SUOsm 334±204mOsm/kgH2O, 24HUOsm 284±253mOsm, 24HUP 0.095±0.087g, ClCr 93.01+15.19ml/min. None of the subjects has ClCr <60ml/min. Six subjects have ClCr 60-89mls/min and were excluded from the study. Mean values of other variables are shown in Table 1.

**Table 1:** Clinical and laboratory characteristics of study population

Variables	Mean (SD)
Age (years)	38.58±11.79
Body mass index (kg/m^2^) both gender	25.51±6.47
Body mass index (kg/m^2^) male	21.74±4.66
Body mass index (kg/m^2^) female	26.98± 6.50
Waist circumference (cm) both gender	82.69±11.36
Waist circumference (cm) male	79.86±10.27
Waist circumference (cm) female	83.78±11.62
Systolic blood pressure (mmHg)	120.4±18.2
Diastolic blood pressure (mmHg)	76.9±10.7
Hemoglobin (g/dl)	12.9±1.6
Serum total cholesterol (mmol/l)	4.41±1.25
Serum LDL-C (mmol/l)	2.87±1.07
Serum HDL-C (mmol/l)	0.97±0.34
Triglyceride (mmol/l)	1.27±0.40
Serum creatinine (mg/dl)	0.88±0.19
Creatinine clearance (mls/min)	93.01±15.19
Spot urine protein (mg/dl)	7.19±18.37
Spot urine creatinine (mg/dl)	147.55±176.43
Spot urine osmolality (mOsm/kgH_2_O)	334±204
24-hour urine volume)ml)	1874± 681
24-hour urine creatinine (mg)	1202.96±315.00
24-hour urine protein (g)	0.095±0.087
24 hour urine osmolality (mOsm)	284±253
SUPCR (mg/mg)	0.082±0.163
SUPOR (mg/dl/mOsm/kgH_2_O)	0.042±0.135
24HUPCR (g/mg)	0.101±0.121
24HUPOR (g/mOsm)	1.410±2.465
SUCOR (mg/dl/mOsm/kgH_2_O)	0.628±0.728
24HUCOR (mg/dl/mOsm)	0.707±0.468

LDL-C=low density lipoprotein cholesterol, HDL-C=high density lipoprotein cholesterol, SUPCR=spot urine protein/creatinine ratio, SUPOR=spot urine protein/osmolality ratio, 24HUPCR=24-hour urine protein/creatinine ratio, 24HUPOR=24-hour urine protein/osmolality ratio, SUCOR= spot urine creatinine/osmolality ratio, 24HUCOR=24-hour urine creatinine/osmolality ratio.

Out of the 130 subjects, 20(14.1%) have proteinuria, while 110(85.9%) did not have proteinuria

Table 2 shows the relationship between proteinuria and selected risk factors in the study population. There was no significant association between proteinuria and different BMI categories, p=0.120. The different WC levels showed no significant association with proteinuria in both males, p=0.737, and females, p=0.457. Similarly, there was no significant association between proteinuria and lipid profiles: cholesterol p=0.263, LDL p=0.943, HDL p=0.637, triglyceride p=0.394.

**Table 2:** Relationship between proteinuria and selected risk factors in study subjects

Variables	Proteinuria Absent (n/%) N=110	Proteinuria Present (n/%) N=20	Chi Square	LHR	P value
BMI <18.5	14(10.3%)	0(0.0)	5.021	0.072	0.120
18.5-24.9	45(33.1%)	12(8.8%)			
25.0-29.9	22(16.2%)	2(1.5%)			
≥30	35(25.7%)	6(4.4%)			
Waist C (cm)					
Male <102	34(91.9%)	2(5.4%)	0.059	0.113	0.737
≥102	1(2.7%)	0(0.0%)			
Female <88	52(53.1%)	10(10.2%)	0.564	0.554	0.457
≥88	28(28.6%)	8(8.2%)			
FSLP (mmol/l)					
Chol T Des (<5.2	91(66.9%)	17(12.5%)	2.669	4.533	0.263
BorderL(5.2-6.2)	12(8.8%)	3(2.2%)			
High (>6.2)	13(9.6%)	0(0.0%)			
LDL Des (<2.6)	56(41.2%)	9(6.6%)	0.075	0.963	0.943
BorderL (2.6- 4.1)	44(32.4%)	8(5.9%)			
High (>4.1)	16(11.8%)	3(2.2%)			
HDL Low (<1)	63(46.3%)	12(8.8%)	0.223	0.225	0.637
High (≥1)	53(39.0%)	8(5.9%)			
TG Des <1.7)	106(77.9%)	20(14.7%)	1.861	3.315	0.394
BorderL (1.7-2.2)	3(2.2%)	0(0.0%)			
High >2.2)	7(5.1%)	0(0.0%)			

LHR=Likelihood ratio, BMI=body mass index, Waist C=waist circumference, FSLP=fasting serum lipid profile, CholT=total cholesterol, Des=desirable BorderL=borderline, LDL=low density lipoprotein cholesterol, HDL=high density lipoprotein cholesterol, TG=triglyceride

Table 3 shows the correlation coefficient (r) of proteinuria with potential risk factors in the study population were: SUCr -0.183, SUOsm -0.225, 24HUV 0.254, SUPCR 0.513, SUPOR 0.366, 24HUPCR 0.682, 24HUPOR 0.702, 24HUCOR 0.440. Negative values mean inverse relationship with proteinuria. The p values were significant, implying that these variables have significant direct association with proteinuria.

**Table 3:** Correlation of proteinuria with variables in study population

Variables	Correlation coefficient	P value
Age	0.000	0.997
Serum creatinine	0.029	0.733
Spot urine protein	0.159	0.066
Spot urine creatinine	-0.183	0.030
Spot urine osmolality	-0.225	0.008
24-hour urine volume	0.254	0.003
24-hour urine creatinine	-0.027	0.753
24-hour urine osmolality	-0.147	0.089
SUPCR	0.513	<0.001
SUPOR	0.366	<0.001
24HUPCR	0.682	<0.001
24HUPOR	0.702	<0.001
SUCOR	0.053	0.543
24HUCOR	0.440	<0.001
ClCr	0.011	0.900
Body mass index	0.046	0.595
Waist circumference	0.362	0.079
Cholesterol	-0.026	0.765
Triglyceride	0.134	0.120
LDL-C	-0.077	0.371
HDL-C	0.057	0.508
Hemoglobin	-0.088	0.306
Systolic blood pressure	-0.014	0.867
Diastolic blood pressure	0.040	0.642

SUPCR=spot urine protein/creatinine ratio, SUPOR=spot urine protein/osmolality ration, 24HUPCR=24-hour urine protein/creatinine ratio, 24HUPOR=24-hour urine protein/osmolality ratio, SUCOR=spot urine creatinine/osmolality ratio, 24HUCOR=24-hour urine creatinine/osmolality ratio, LDL-C=low density lipoprotein cholesterol, HDL-C=high density lipoprotein cholesterol,

Conversely, there was no significant correlation between proteinuria and age, serum creatinine, SUP, 24HUCr, 24HUOsm, SUCOR, ClCr, BMI, WC, total cholesterol, triglyceride, LDL, HDL, hemoglobin, SBP, DBP.

Multivariate linear regression analysis of the potential predictors of proteinuria is shown in Table 4. High 24HUV, high SUPCR, high 24HUPCR, high 24HUPOR, low SUPOR and low 24HUCOR were predictors of proteinuria in this study.

**Table 4:** Multivariate linear regression analysis showing risk factors for proteinuria in study population

Variable	Beta	t	p value
Spot urine creatinine	0.000	0.000	1.000
Spot urine osmolality	-0.045	-0.812	0.418
24-hour urine volume	0.118	2.173	0.032
SUPCR	0.448	4.483	<0.001
SUPOR	-.345	-3.339	0.001
24HUPCR	0.381	6.813	<0.001
24HUPOR	0.676	7.674	<0.001
24HUCOR	-0.315	-3.745	<0.001

Model Summary R=0.861, R^2^=0.742, Standard error of estimate=0.046, p<0.001.

SUPCR=spot urine protein/creatinine ratio, SUPOR=spot urine protein/osmolality ratio, 24HUPCR=24-hour urine protein/creatinine ratio, 24HUPOR=24-hour urine protein/osmolality ratio. 24HUCOR=24-hour urine creatinine/osmolality ratio.

## Discussion

This study showed that the predictors of proteinuria were high 24HUV, high SUPCR, high 24HUPCR, high 24HUPOR, low SUPOR, and low 24HUCOR in subjects without impaired renal filtration function in Owerri, Nigeria.

The finding of 24HUV as a predictor of proteinuria in this study tends to suggest that kidney damage associated with proteinuria will also give rise to passage of increased urine volume. A study reported increasing urine volume associated with decreasing renal function [11]. The study, however, did not evaluate the association of urine volume with proteinuria, but urine volume in reduced renal function defined only by reduced renal filtration function. Nevertheless, our finding does not agree with earlier study in which kidney disease was shown to be associated with fluid retention and reduced urine volume [12].

However, glomerular disease with proteinuria may later affect the interstitial compartment and may lead to distal tubular damage, some with attendant nephrogenic diabetes insipidus that is associated with polyuria [13, 14].

Low SUCr was associated with proteinuria in this study. This agrees with established fact that urine creatinine decreases as kidney damage progresses [15]. However, multi-linear regression analysis showed SUCr was not a predictor of proteinuria. SUCr is known to vary over 24 hours of the days and may not indicate quantitatively the presence of renal damage [16].

Low SUOsm was, as found in this study, significantly associated with proteinuria. Further analysis, however, showed it was not a predictor of proteinuria. Low SUOsm may suggest a low capacity to regulate and concentrate urine by the kidney. This finding is in consonance with the report from studies which showed that reduced ability to concentrate urine occurred as kidney damage progressed [11, 17].

Both SUPCR and SUPOR were predictors of proteinuria in this study. They are indices of 24-hour urine protein estimation, and expectedly, should reflect proteinuria.

We found that both 24HUPCR and 24HUPOR were predictors of proteinuria in this study. They are not routine screening tools in clinical practice. Literature search did not reveal any studies in which they were used in evaluating renal function.

Our study also noted that 24HUCOR was a negative predictor of proteinuria. Simply put, as proteinuria increased, 24HUCOR decreased. From literature search, we did not find any study that compared 24HUCOR in subjects with proteinuria and those without proteinuria.

No association was found between age and proteinuria. However, Agbaji et al [18] reported association of age with CKD. In their study, the study population consisted of HIV subjects, whereas ours, here, was a non-HIV population. In addition, CKD in their study design was defined by GFR, in contrast to our study which was on proteinuria without impaired renal filtration function.

We did not find any association between cholesterol, triglyceride, LDL-C, HDL-C, with proteinuria. A study, however, reported high cholesterol and high triglyceride as predictors of CKD in HIV subjects, contrasting with our study in non-HIV population [19, 20].

Hemoglobin was not associated with proteinuria in this study. In contrast, anemia was associated with CKD in one study [21].

This study also showed that there was no association between SBP, DBP and proteinuria. In contrast, some studies found SBP and DBP as predictors of CKD [20–23]. Our study exclusion criteria removed those subjects that were hypertensive. Most of these reference studies used GFR to define renal damage, whereas our study used only proteinuria to denote renal damage, without impairment of renal filtration function.

## Conclusion

The risk factors of proteinuria in this study population included high 24HUV, high SUPCR, high 24HUPCR, high 24HUPOR, low SUPOR and low 24HUCOR. Further search should explore the relationship between urine creatinine and urine osmolality, and how this relationship may affect progression of kidney damage with or without impaired renal filtration function. Limitations: The study population was small. A larger study population size would have been better.

### What is known about this topic

- Proteinuria is a marker of renal damage;
- Some risk factors of proteinuria have been identified;
- Kidney damage is commonly identified with reduced glomerular filtration rate (GFR).

### What this study adds

- When GFR alone is used to screen for kidney damage, many subjects who have kidney damage but do not have impaired GFR may be missed out;
- Risk factors of proteinuria identified in this study are a mirror of kidney damage in patients without impaired renal filtration function in a general population;
- Some of these risk factors should further be explored to determine what relationship urine creatinine may have with urine osmolality.
